# The Smart City Active Mobile Phone Intervention (SCAMPI) study to promote physical activity through active transportation in healthy adults: a study protocol for a randomised controlled trial

**DOI:** 10.1186/s12889-018-5658-4

**Published:** 2018-07-16

**Authors:** Anna Ek, Christina Alexandrou, Christine Delisle Nyström, Artur Direito, Ulf Eriksson, Ulf Hammar, Pontus Henriksson, Ralph Maddison, Ylva Trolle Lagerros, Marie Löf

**Affiliations:** 1 Department of Biosciences and Nutrition, Karolinska Institutet, Group MLÖ, 141 83 Huddinge, Sweden; 2 Healthy Active Living and Obesity (HALO) Research Group, Children’s Hospital of Eastern Ontario Research Institute, University of Ottawa, 401 Smyth Road, Ottawa, ON K1H 8L1 Canada; 3 Centre for Behaviour Change, University College London, Alexandra House, 17-19 Queen Square, London, WC1N 3AR UK; 4 Trivector Traffic, Barnhusgatan 16, 111 23 Stockholm, Sweden; 5 Institute of Environmental Medicine, C6, Biostatistics, Karolinska Institutet, 210, 171 77 Stockholm, PO Sweden; 6 Institute for Physical Activity and Nutrition, School of Exercise and Nutrition Sciences, Deakin University, 221 Burwood Highway, Burwood, Melbourne, VIC 3125 Australia; 7 Clinical Epidemiology Unit, Department of Medicine, Karolinska Institutet, Eugeniahemmet T2, 171 76 Stockholm, Sweden; 8 Clinic of Endocrinology, Metabolism and Diabetes, Department of Medicine, Karolinska University Hospital, 141 86 Huddinge, Sweden

**Keywords:** Accelerometer, Active transport, Application, Behaviour change, mHealth, Smartphone, Physical activity, Walkability

## Abstract

**Background:**

The global pandemic of physical inactivity represents a considerable public health challenge. Active transportation (i.e., walking or cycling for transport) can contribute to greater total physical activity levels. Mobile phone-based programs can promote behaviour change, but no study has evaluated whether such a program can promote active transportation in adults. This study protocol presents the design and methodology of The Smart City Active Mobile Phone Intervention (SCAMPI), a randomised controlled trial to promote active transportation via a smartphone application (app) with the aim to increase physical activity.

**Methods/design:**

A two-arm parallel randomised controlled trial will be conducted in Stockholm County, Sweden. Two hundred fifty adults aged 20–65 years will be randomised to either monitoring of active transport via the TRavelVU app (control), or to a 3-month evidence-based behaviour change program to promote active transport and monitoring of active travel via the TRavelVU Plus app (intervention). The primary outcome is moderate-to-vigorous intensity physical activity (MVPA in minutes/day) (ActiGraph wGT3x-BT) measured post intervention. Secondary outcomes include: time spent in active transportation measured via the TRavelVU app, perceptions about active transportation (the Transport and Physical Activity Questionnaire (TPAQ)) and health related quality of life (RAND-36). Assessments are conducted at baseline, after the completed intervention (after 3 months) and 6 months post randomisation.

**Discussion:**

SCAMPI will determine the effectiveness of a smartphone app to promote active transportation and physical activity in an adult population. If effective, the app has potential to be a low-cost intervention that can be delivered at scale.

**Trial registration:**

ClinicalTrials.gov NCT03086837; 22 March, 2017.

## Background

Promoting physical activity is considered the best buy in public health [1]. The health benefits of regular physical activity are considerable [2–4] and occur even with modest improvements in activity levels [5]. Despite these benefits, 31% of the global population are physically inactive [6], therefore, low-cost interventions that can be delivered at scale are a public health priority [7]. One way to increase physical activity at the population level is to make it easier to integrate in individuals’ daily life routines [8]. Active transportation (i.e., any self-propelled mode of transportation to get from one place to another such as walking or biking to work, school, or running errands) has been shown to be a feasible approach (i.e., more convenient and affordable in comparison with planned exercise/recreational activity) for increasing total physical activity levels [9]. Thus, active transportation is an optimal target for physical activity promotion with substantial health benefits at a population level [10–12]. For instance, active transportation has been inversely associated with all-cause mortality, cardiovascular disease, falls, impaired mental health, type 2 diabetes and obesity [12–14]. Cycling or walking to work was associated with a 50% lower risk for type 2 diabetes when compared to car, taxi or motorcycle transportation [14]. Furthermore, walking or cycling for transportation was associated with a lower body mass index (BMI) when compared to those who went by car, taxi or motorcycle [13].

The ubiquitous use of smartphones offers considerable possibilities for the promotion of physical activity, particularly active transportation. In-built (native) sensors in smartphones, such as accelerometers, gyroscopes and global positioning systems (GPS) can be used to track people’s travel modes (e.g., walking, cycling, and motorised transportation) and provide information such as speed, duration and length of travel behaviour. Mobile health (mHealth, including smartphones), interventions are possible to deliver at scale, and have a number of benefits over traditional intervention approaches. mHealth programs can be delivered anywhere at any time, are interactive, and can be tailored to meet people’s needs. Compared to traditional face-to-face counselling such programs are less time-consuming and cheaper to deliver [15]. Recent systematic reviews have concluded that mHealth programs may be useful for achieving changes in behaviour such as physical activity, and for weight loss [16–18]. In the context of active transportation, smartphones can be used to provide real-time measures of travel behaviour and deliver a behaviour change intervention. One previous study used a smartphone app to promote active transport at a university campus; however, it was part of a multi-strategy campaign, thus, the effect of the app itself could not be evaluated [19, 20]. To the best of our knowledge no study has evaluated whether a smartphone application (app) can promote active transportation.

### Aims

The aim of this paper is to report the study design and methodology of the Smart City Active Mobile Phone Intervention (SCAMPI). The primary aim of SCAMPI is to examine if an app can promote active transportation to increase physical activity in adults. Secondary aims are to determine the effects of SCAMPI on active transportation, attitudes towards active transport and health related quality of life.

### Hypothesis

The primary hypotheses are that compared to controls, the intervention group will spend more time (minutes per day) on moderate-to-vigorous intensity physical activity (MVPA) immediately post-intervention and at the 6 month follow-up. Secondary hypotheses are that the intervention group will spend more time on active transportation (cycling or walking), that the intervention effect will be larger among those who at baseline perceive their neighbourhood walkability as low and that attitudes towards active transport will improve more compared to the controls. We further hypothesize that the intervention group will report improved health related quality of life at the end of the intervention and at the 6 month follow-up.

## Methods

### Study design

**Fig. 1:**
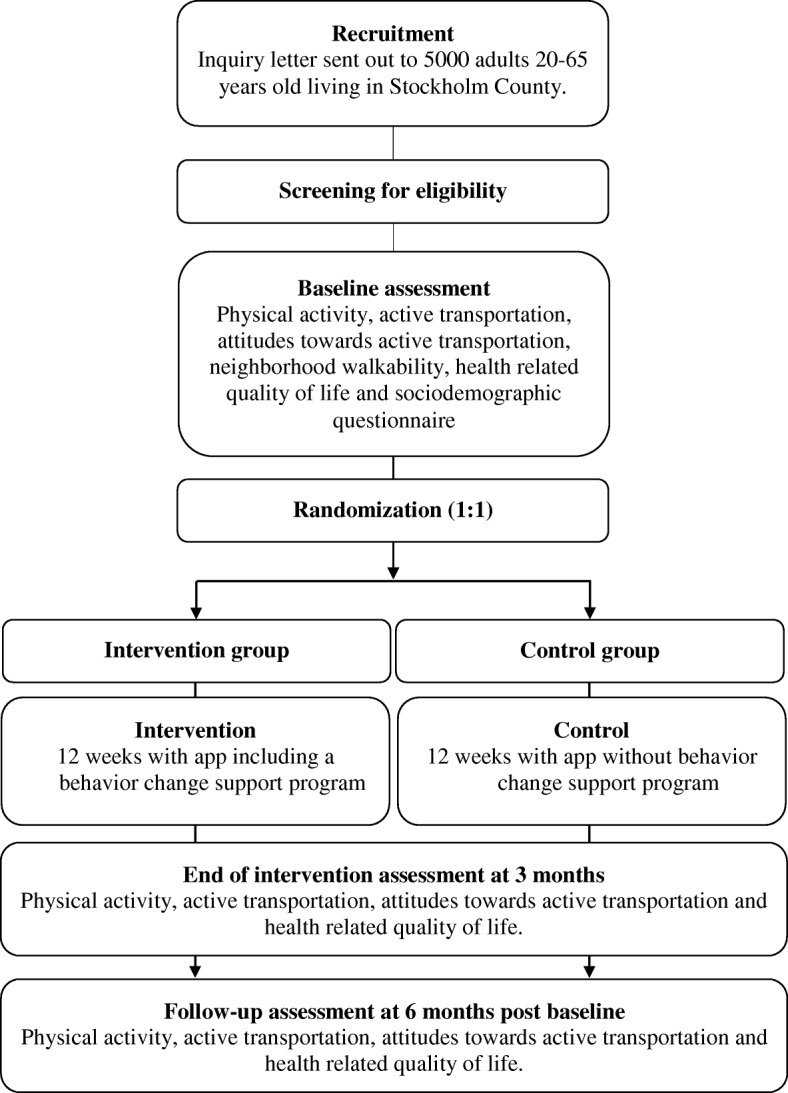
Flow-chart of the SCAMPI trial design

SCAMPI is a two-arm parallel randomised controlled trial (RCT) (1:1 ratio) conducted in Stockholm County, Sweden, with assessments at baseline, post intervention at 3 months and 6 months post randomisation (see Fig. 1 for study outline). This study protocol follows the SPIRIT 2013 statement [21, 22] and the intervention is described according to the CONSORT – EHEALTH checklist [23].

### Participants and recruitment

Participants are recruited from a random sample of 5000 adults (aged 20–65 years) living in Stockholm County drawn from the population register at Statistics Sweden. Eligible participants are 20–65 years old, able to understand Swedish well enough to fully understand the study aims and consent to participate, and have access to a smartphone. Those who are unable to perform MVPA (i.e. brisk walking or cycling) will be excluded.

Potential participants are sent an inquiry letter by Statistics Sweden to their home address providing them with written information about the study. Participants register their interest via the study website, send in a response letter by post or contact the research group directly by email or telephone. Those who do not respond to the inquiry letter (including the two reminders after 2 and 4 weeks, respectively) will remain anonymous to the research team. Once informed consent is obtained, baseline measures are collected. Participants are asked to fill in a web-based questionnaire with questions regarding perceived neighbourhood walkability, attitudes towards active transportation, health related quality of life, self-reported physical activity level and socio-demographic data. Thereafter, participants are asked to wear an accelerometer to measure physical activity for seven consecutive days. Participants are also asked to download an app free of charge on to their smartphone, TRavelVU (Trivector AB ©), which passively captures travel behaviour for the same seven days as the accelerometer is worn. The app is compatible with both iOS (version 8.4 or higher) and Android (version 4.4 or higher) smartphones.

### Randomisation and blinding

Upon completion of baseline measures, participants are randomised to the intervention or the control group in a 1:1 ratio. A computer-generated random allocation sequence list (in blocks of two) is generated in Stata 13 (StataCorp LP, College Station, Texas, USA). To ensure allocation concealment to study personnel until group allocation, the allocation list is monitored by a member of the research group with no participant contact. After randomisation, participants receive an email containing information about their group allocation. Assessors of the primary and secondary outcomes are blinded to group allocation; however, due to the design of the study participants are not blinded to their allocation.

### Control group

The control group participants are encouraged to continue with their normal routines and to monitor their daily travels in the TRavelVU app. The app passively collects data on the mode of travel, duration and speed for 6 months, but no behaviour change content is provided. Upon completion of the trial, participants from the control group will have access to TRavelVU Plus, see below, if they wish to.

### Intervention

#### Overall

The overall aim of the SCAMPI intervention is to increase physical activity levels by promoting active transport. Once allocated to the intervention group, participants receive an email with a user manual for the TRavelVU Plus app. This app differs from the TRavelVU app as it includes a 3-month behaviour change program promoting active transportation. Features of the TRavelVU Plus app (i.e., messages (sent as push notifications), goal setting and feedback functions) are activated and start with a welcome message. The message includes a goalsetting function where participants set their first weekly goal for active transportation, for example 5 h. As guidance, they are informed of the recommendations for adults of 150 min of MVPA per week, but are encouraged to set a goal based on their current activity level. Feedback on the goal is provided in different formats during the week. At the end of the week, participants are able to adjust their goal for the following week. After 3 months, the features of TRavelVU Plus disappear; however, participants are encouraged to keep registering their travels in the TRavelVU app until after the 6-month follow-up assessment.

#### Development and content

##### The TRAvelVU app

**Fig. 2:**
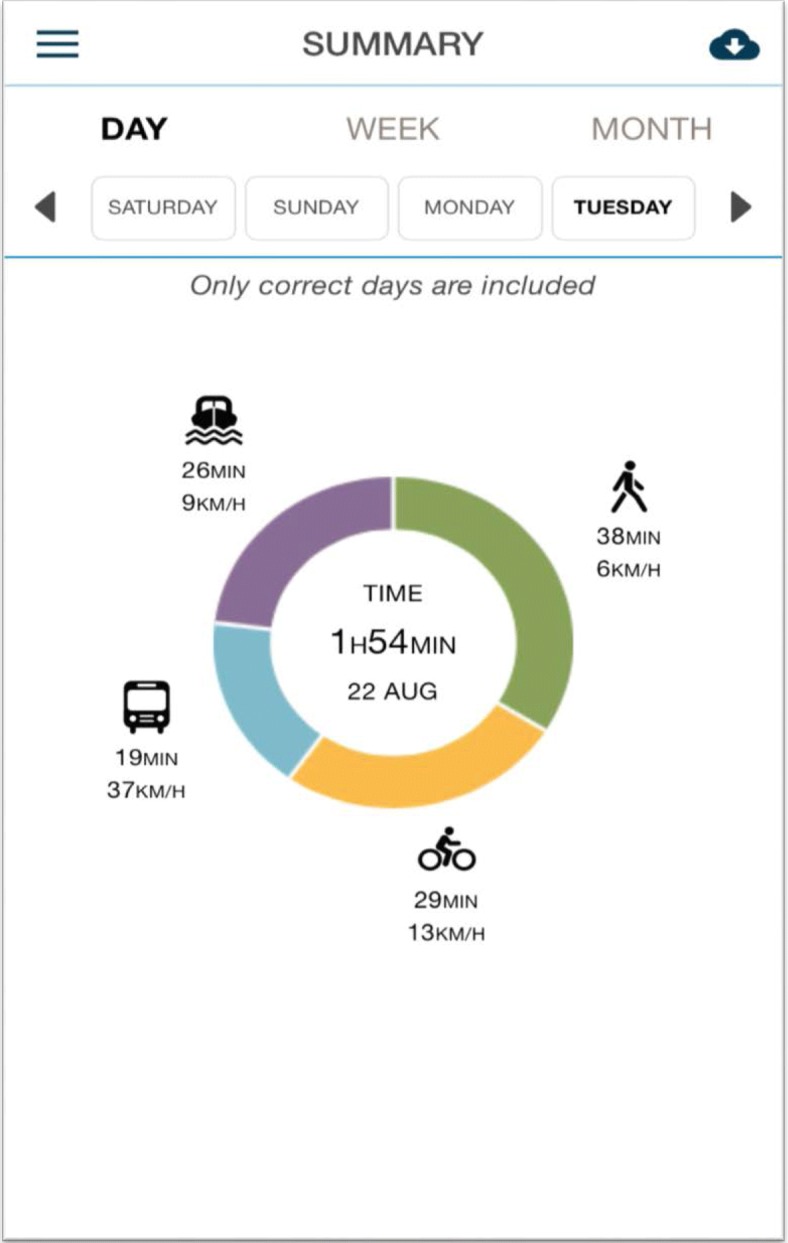
Screenshot of daily summary of travel behaviours in the TRavelVU app

TRavelVU, an automatised and objective assessment of travel behaviour was developed by Trivector AB ©, a company with expertise in sustainable and active transportation. Once downloaded, the TRavelVU app continuously and passively (without manual input) collects data in real time on location and travel speed (GPS). When the user is in motion, the app starts to send GPS data to the server, and these data are transferred into speed and acceleration profiles. The analysed data are then immediately sent back to the app where participants can view their journey (distance, route and suggested mode of transport). The users enter data about their destinations in the app and edit modes of transportation and start/stop times of trips if these have been registered incorrectly. Once a location has been added to the app by the user (e.g., “home” or “office”), TRavelVU remembers this and automatically suggests this destination for future trips. Algorithms are based on a fuzzy logic approach on a number of different variables (e.g., mean speed and percentile speed), to identify the most likely modes of transportation for trip segments [24, 25]. The app has been developed with the aim of minimising battery consumption. All participant data are anonymous and linked to a unique identification number. Using these data, TRavelVU provides detailed information on the participant’s travel behaviour. Fig. 2 shows a screenshot of a daily summary of travel modes for a user in TRavelVU.

##### Behaviour change program (the TRavelVU plus app)

**Fig. 3:**
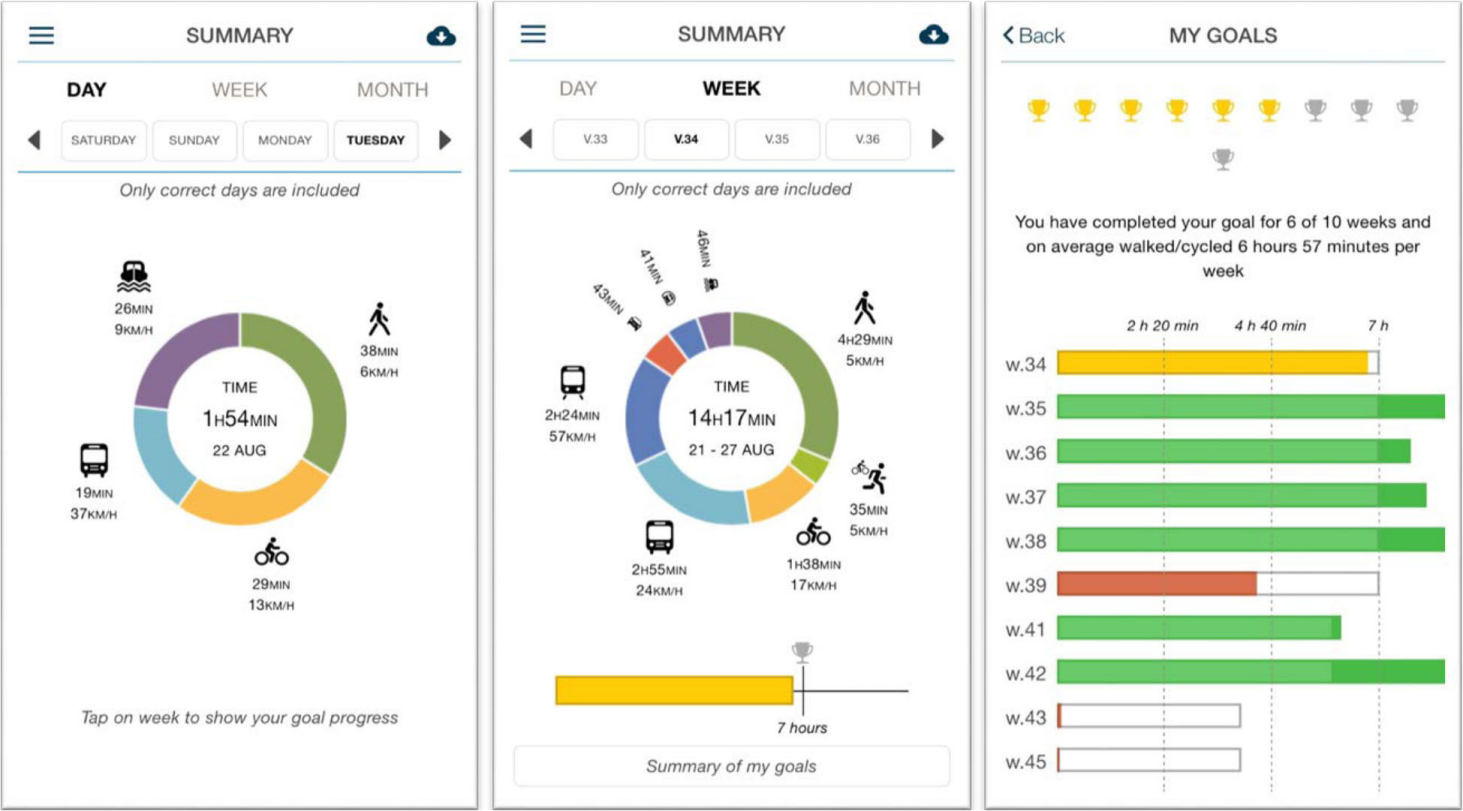
Screenshots of daily and weekly summaries of travel behaviours and achievements related to set goals

The behaviour change program in the TRavelVU Plus app was developed by Trivector AB © and researchers with experience in behaviour change interventions (obesity, physical activity and transport). The intervention is grounded in Social Cognitive Theory [26] and Social-Ecological [27] principles. To improve the reporting of the content in behaviour change interventions the behaviour change technique taxonomy is recommended [28]. Thus, key behaviour change techniques known to promote physical activity behaviour are included in the app’s features and text messages (e.g., general information about physical activity and active transport, information regarding consequences, barrier identification, specific goal-setting strategies and functions, self-monitoring and feedback on performance) [28]. See Table 1 for theoretical domains, behaviour change techniques and examples of messages. We also considered recommendations from previous research regarding message linguistics (e.g., grammar, spelling, and positivity), dosage, length as well as timing of delivery [29, 30]. Thus, participants receive 4–6 messages per week that contain no more than 200 characters. The program consist of a core set of messages scheduled to be sent at fixed time points throughout the intervention that all participants receive. The messages include practical tips, general information and strategies for behaviour change. The messages can be accessed at any time throughout the intervention and are organised so that the content follow the study timeline. Thus, in the beginning of the intervention messages provide information about features within the app and strategies for goalsetting, and later on the messages focus on past successes and strategies for sustained behaviour changes. The goalsetting feature allows participants to set weekly individual goals for active transportation (i.e., walking and cycling). Feedback is provided in graphical format, where participants can follow their daily and weekly achievements and as a summary of all previous goals (see Fig. 3). During the week a coloured symbol (red, yellow, or green) guides the participants if their present achievement thus far in time spent in active transportation is enough to reach their goal or if greater efforts are needed. Additionally, at the end of each week a feedback message is sent out, which is tailored and varied according to participants’ goals and level of goal achievement in hours and minutes (i.e., ≥100%, ≥70, < 70% or no goal was set).

**Table 1:** TDF’s and BCT’s used in the TRavelVU (control) and TRavelVU Plus (intervention) apps

TDF	BCT’s	Example of a message in the TRavelVU Plus app
Knowledge	Information about health consequences Instructions on how to perform the behaviour Provide information about the behaviour Information about antecedents	You do not need professional equipment to get started. Find your old bike, borrow one or buy one secondhand. Find out where you can park your bike at work and decide your start date!
Skills	Behavioural practice/rehearsal Habit formation Habit reversal Behaviour substitution	Do you commute using public transport? Why not get off at an earlier stop and walk the rest of the way?
Social/professional role and identity	Identification of self as a role model	Do you think someone close to you would benefit from being more physically active? Remember, you’re a role model for others! Be the first to change to a more active lifestyle!
Beliefs about capabilities/Optimism	Verbal persuasion to boost self-efficacy Mental rehearsal of successful behavior Focus on past success Verbal persuasion to boost self-efficacy	It is motivating to succeed in changing a habit. Start by setting a challenging but achievable goal, for example to cycle to work at least once a week instead of taking the car.
Beliefs about consequences	Information about social and environmental consequences Information about health consequences Pros and cons Comparative imagining of future outcomes Material reward (behaviour) Reward (outcome)	Cyclists contribute to cleaner air and less noise. Reduced car traffic makes the roads safer - safer roads promote cycling.
Reinforcement	Reward (outcome) Reward	Are there others in your household or at work who want to walk or cycle more? Set up a common goal to get started. Why not with a reward to do something fun together after you have walked or cycled 50 km?
Goals	Goal setting (behaviour) Problem solving Review behaviour goals Action planning Barrier identification Commitment	Is it possible for you to increase the time you spend walking and cycling? Set up achievable goals and check your progress (in the app) every day.
Intentions	Action planning Commitment	Is it difficult to get started with active transport? Plan your start date and stick to your plan, then take it one day at a time. Remember to set up achievable goals.
Social influences	Social support (practical) Social support (emotional) Social comparison Social support	Limited time is one reason why 50% of Swedish adults are physically inactive. Active transport can facilitate physical activity when there is no time for exercise. How can you create time for more active transport in your life?
Emotion	Reduce negative emotions Social support (emotional)	How are you doing? Changing habits can be challenging. Make sure to focus on what works.
Behavioural regulation	Self-monitoring of behaviour (outcome)^a^ Self-monitoring of outcome(s) of behaviour Feedback on behaviour^a^	Keeping track will help you to reach your goal. Go to *Summary* (in the app) to track your trips during the day and throughout the week. Is there any part of your travel that can be replaced with active transport?

The behaviour change taxonomy (v1) [28] where used to list theoretical domains frameworks (TDF’s) and behaviour change techniques (BCT’s) used in the TRavelVU and TRavelVU Plus apps

^a^Features present in the TRavelVU app availible to the control group (e.g., feedback as summary of active transportation (not in relation to a set goal))

##### Pilot study

Prior to undertaking the full RCT a pilot study was conducted to assess the usability and content of TRavelVU Plus (features and messages) and to obtain other relevant information that could be integrated into the final version of the app. A convenience sample of 13 adults (7 women and 6 men, mean age 40 years) were recruited. The pilot participants were asked to use the app for two weeks and then complete questions regarding app usability, how they perceived the specific features in TRavelVU Plus, as well as the content and dose of the messages. Participants were also able to give general comments on the functions they did or did not appreciate, as well as to provide suggestions for improvements. As we were unable to test all messages via the app in the pilot study, semi-structured interviews were conducted via telephone with 8 of the pilot participants (3 woman and 5 men, mean age 36 years). During these interviews the entire message library (87 messages) were read aloud. Pilot participants were asked how they perceived each message and were encouraged to provide suggestions on how the message could be improved. The most appreciated features within the app were the goalsetting and self-monitoring functions. The battery consumption was reported as a limitation. Some participants thought it had been unclear how to edit trips and travel modes in the app. Another reported limitation was the inconvenience of having to specify all locations to be able to accept the registered day as valid. The messages were generally perceived as acceptable and relevant as well as delivered in a suitable dosage. However, more individually tailored messages were requested. Suggestions to add a step count function was also highlighted. Participants that were already physically active asked for more challenging messages and suggestions for exercise.

Based on the feedback from the pilot study, the app was revised. The updated app allows participants to accept a registered day as valid even if locations have not been specified (e.g., if one stopped to talk to a neighbour for 3 min during a longer walk). In regards to the manual the participants receive, we clarified how to edit trips manually and that greater battery consumption will occur when using the app. Furthermore, within the manual the study aim was highlighted (i.e., to increase physical activity through active transportation and not through exercise) so that the expectations of the study would be clear for the participants. After the pilot study and discussions within the research group minor changes were made to the wording of the messages and the number of messages were condensed from 87 to 61 to conform to the number needed during the intervention.

### Sample size and power considerations

A total of 200 participants (100 in each group) completing follow-up will provide 80% power (α = 0.05) to detect a 10 min difference in MVPA (e.g., 35 versus 45 min/day) assuming a standard deviation of 25 min [31]. Assuming about 20% drop-out rate between baseline and follow-up 250 participants will be enrolled in the study [32]. We will continue to sample until this goal has been reached. Based on our previous experience we estimate that 5000 randomly selected subjects will provide us with the sample requested when considering loss to follow-up and subgroup analyses [33].

### Outcomes and measures

The primary outcome is MVPA post intervention (i.e., 3 months post randomisation). Secondary outcomes include active transportation, neighbourhood walkability, health related quality of life and attitudes towards active transportation. Outcomes are assessed at baseline and at 3 and 6 months post randomisation. See Table 2 for an overview of all assessments. Unexpected and adverse events are voluntarily reported by participants to the research team and will be reported.

**Table 2:** Outcomes and other measures collected at the different time points

		Baseline	3 months	6 months
*Assessments*	*Measure*			
MVPA, minutes/day	ActiGraph wGT3x-BT	x	x	x
Leisure and work time physical activity^a^	Questionnaire	x	x	x
Travel behaviour (active transportation)	Smartphone application (TRavelVU)	x	x	x
Health related quality of life	RAND-36	x	x	x
Attitudes towards active transportation	Transport and physical activity questionnaire (TPAQ)	x	x	x
Neighbourhood walkability	Neighbourhood environment walkability scale (NEWS)	x		
Sociodemographic factors^b^	Questionnaire	x		

Abbreviations: *MVPA* moderate-to-vigorous physical activity

^a^Two questions in the web-based questionnaire are used to assess leisure and work time physical activity [34]

^b^Occupational status, area of residency, type of housing and access to a bicycle were also repeated at the 3 and 6 month follow-ups

#### Physical activity and MVPA

Leisure time and work related physical activity were measured with two questions included in the web based questionnaire [34]. To provide an objective assessment of physical activity an ActiGraph wGT3x-BT accelerometer (https://www.actigraphcorp.com/) are sent by mail to participants’ homes for them to wear for seven consecutive days. The accelerometer is worn on the waist at the mid-axillary line (on the right side), lying on the iliac crest during waking hours. The participants are asked to report all time points when the accelerometer was not worn in a diary accompanying the accelerometer (e.g., when taking a shower). The accelerometers are set to collect data at 90 Hz. The collected data will be processed in accordance with our previous procedures [35] following the accelerometer data processing criteria (e.g., regarding cut-points for MVPA, number of valid days) published in our recent review [36].

#### Active transportation

Active transportation (mode and duration in hours and minutes) will be assessed using GPS data from the app, using only registrations that participants have marked as valid. In addition, attitudes towards active transportation will be assessed using the psycho-social measures in section B of the validated Transport and Physical Activity Questionnaire (TPAQ) [37]. Participants are asked to what extent they agree (i.e., strongly agree, somewhat agree, neither agree nor disagree, somewhat disagree, or strongly disagree) to 20 statements regarding walking (10 statements) and cycling (10 statements) as transportation. Examples of statements are: “Walking/cycling to travel from place to place is something I do automatically without really thinking about it.”, “Walking/cycling for travel is enjoyable.” and “It is possible for me to walk/cycle for travel”.

#### Health related quality of life

Health related quality of life will be assessed using the commonly used RAND-36 questionnaire [38, 39]. The RAND-36 consists of 36 items and measures of perceived health across eight domains using a 0–100 scale: 1) physical functioning; 2) bodily pain; 3) role limitations caused by physical health problems; 4) role limitations caused by emotional problems; 5) general mental health; 6) social functioning; 7) energy/fatigue and (8) general health [40].

#### Neighbourhood walkability

Perceived neighbourhood walkability will be assessed at baseline using the Neighbourhood Environment Walkability Scale (NEWS) questionnaire [41–43]. The NEWS scales to assess neighbourhood attributes used in SCAMPI are: (1) residential density; (2) land use mix – diversity; (3) land use mix – access; (4) street connectivity; (5) infrastructure and safety for walking; (6) aesthetics; (7) traffic safety; (8) safety from crime; (9) streets not having many cul-de-sacs and (10) physical barriers to walking. To calculate a perceived walkability index for each participant we will use: residential density, street conductivity and land use mix as previously described [42].

#### App usability and quality assessment

To assess the participant’s use of the app we will collect data on the number of registered days (i.e., days the participants marked as valid in the app) for both groups. For the intervention group the number of weekly goals that were set will be assessed and if goals were met, based on the feedback messages delivered. To assess usability and perceived quality of the TRavelVU Plus app, semi-structured interviews will be conducted in a sub-sample of participants who complete the intervention.

#### Sociodemographic factors

At baseline, participants will complete questions regarding sociodemographic data e.g., sex, age, country of birth, educational attainment, weight and height, and family structure. At follow-up only questions that may have changed and are of relevance for the purpose of the study are repeated, such as if the participant has moved, occupational status or if the participant has access to a bike.

### Statistical analyses

Trial evaluation will be performed with linear mixed model analyses using intention to treat principles. In the statistical models we will use time as a categorical variable (0, 3 and 6 months) with time 0 as a reference group and include an interaction between group and time. The dependent variable is post intervention scores for MVPA (i.e., minutes/day) and the secondary outcomes (e.g., active transportation (minutes/day)). Three-way interactions will be analysed in order to evaluate if the intervention effect was moderated by any of the baseline covariates (e.g., age, sex, educational attainment, neighbourhood walkability or health related quality of life). Missing values on baseline covariates and on follow-up assessments will be imputed simultaneously using multiple imputation with chained equations [m (number of imputations) = 20] [44] taking into account variables of importance for predicting the missing values (e.g., age, sex, country of birth, educational attainment, occupation, season for intervention (i.e., autumn or spring) etc.). Continuous variables will be imputed using predictive mean matching (5 nearest neighbours). Binary variables will be imputed using logistic regression, and multi-categorical variables will be imputed using ordinal logistic regression (if ordered) or multinomial logistic regression (if unordered). Descriptive data over adherence to the intervention will be presented. Per protocol analyses examining effects related to adherence to the intervention (e.g., number of registered days in the app, goals set and achieved) will be conducted using linear mixed models adjusting for age, sex, country of birth (Sweden/not Sweden), educational attainment, occupation and season for intervention. As in the primary analysis, time, group and time-group-interactions will also be included. Sensitivity analyses concerning deviations from the missing at random assumption will be performed using delta-adjustment [45]. Statistics Sweden will provide summary statistics for responders and non-responders regarding age, sex, country of birth, education level and area of residence. All *p*-values < 0.05 (two-sided) will be regarded as statistically significant.

### Trial status

In June 2017, final modifications to TRavelVU Plus were made and in August 2017 the app content and usability was tested in a pilot study, with minor changes to the app being made thereafter. Recruitment of participants, baseline assessments and randomisation of participants started in September 2017 and will continue until April 2018. Outcome assessments at the end of the intervention will be finalised by July 2018. Follow-up assessments (six months after randomisation) will be finalised in October 2018.

## Discussion

The SCAMPI trial aims to evaluate the effectiveness of a 3-month behaviour change program delivered via a smartphone app to promote active transportation (i.e., walking and cycling) in adults aged 20–65 years. Active transportation has great potential to increase physical activity levels and thus influence the health of many people; however, interventions targeting travel behaviours are few. To the best of our knowledge, SCAMPI is the first study to assess a smartphone app to deliver such an intervention.

The SCAMPI study has many strengths, including the randomised controlled design and the relatively large population based sample. This design will enable us to assess travel behaviour and intervention effectiveness in a heterogeneous sample representing both urban and non-urban areas in the Stockholm County. Furthermore, the use of objective measures for both physical activity (accelerometers) and travel behaviour (TravelVU app) will provide unique data extending previous research on this topic that have been limited to self-reported data [46]. The novelty of SCAMPI is the development of a smartphone app including a behaviour change program to support participants to engage in active transportation. To date, we found one prior study using an app to promote active transportation as part of a larger campaign; however, the web based app in that study targeted a selected population and used a non-randomised design that did not enable the effectiveness of the app to be assessed [20]. The urgent need to find effective solutions to decrease the alarmingly high levels of sedentary behaviour among adults makes the focus on active transportation valuable. Active transportation may be an attractive option for people to accumulate physical activity time in their everyday life, who otherwise would be inactive. The TRavelVU Plus app therefore has the potential to make a difference on health outcomes for those in greatest need of this change.

The behaviour change program is anchored in the Social Ecological Theory and the Social Cognitive Theory. A theoretical approach not only guides the intervention developers to appropriate trial content, but has the advantage of making the intervention easier to replicate if found effective [47]. Theoretical frameworks are also recommended to make interventions more effective [47, 48]. A further strength of the behaviour change program is the use of several behaviour change techniques from different theoretical domains [28]. To include a variety of intervention functions and behaviour change techniques have been associated to intervention success [49, 50]. Specifically, ‘self-monitoring of behaviour’ and ‘intention formation’ have proven valuable in interventions that promote active transport, such as cycling and walking [49, 51]. Furthermore, the intervention was developed using key psychological theories and behaviour change strategies known to positively influence physical activity [52, 53].

Limitations of the trial are that we were unable to individually tailor messages due to budget constraints; a feature that has been associated with higher participant engagement [30]. While the objective assessment of travel behaviour in both groups is a strength of this study, participants in the control group will be aware of their travel behaviours through the TRavelVU app, which may have an effect on their behaviour and thus may dilute the effect of the intervention. Finally, a technical consideration that may be a limitation for some users is battery consumption on their smartphones when using the app.

We chose to recruit our population from a random sample of the general population, using letters sent out by Statistic Sweden, which we have successfully used previously [32, 54]. We believe that we thereby obtain a heterogeneous sampling frame of participants, enabling us to examine the intervention effect among participants with various characteristics. It is possible that this study attracts those who are already physically active and thus the intervention effect may be reduced. However, with the chosen design we have the opportunity to reach a large sample and to test the usability of the app in an appropriate manner before extending the use of the app to other populations. In the future, possible arenas for implementation of the app are municipalities wanting to improve public health in the general population. Thus, we are evaluating the app in the same context as it will be delivered.

Active transportation is cheap, easy-to-use and can become a daily habit for many people. Thus, if the TRavelVU Plus app is effective it has potential to offer a low-cost intervention that can be delivered at scale to promote active transportation. Active transportation can significantly contribute to increasing physical activity levels and thus improve the health of many and thereby decrease related societal health care costs.

## Abbreviations

App: Application
BCT: Behaviour change techniques
mHealth: Mobile health
MVPA: Moderate-to-vigorous intensity physical activity
NEWS: Neighbourhood environment walkability scale
SCAMPI: The Smart City Active Mobile Phone Intervention
TDF: Theoretical domain framework
TPAQ: Transport and physical activity questionnaire
